# Host-associated helminth diversity and microbiome composition contribute to anti-pathogen defences in tropical frogs impacted by forest fragmentation

**DOI:** 10.1098/rsos.240530

**Published:** 2024-06-12

**Authors:** Wesley J. Neely, Kassia M. C. Souza, Renato A. Martins, Vanessa M. Marshall, Shannon M. Buttimer, Ananda Brito de Assis, Daniel Medina, Ross D. Whetstone, Mariana L. Lyra, José Wagner Ribeiro, Sasha E. Greenspan, Célio F. B. Haddad, Luciano Alves dos Anjos, C. Guilherme Becker

**Affiliations:** ^1^ Department of Biology, The University of Alabama, Tuscaloosa, AL 35487, USA; ^2^ Department of Biology, Texas State University, San Marcos, TX 78666, USA; ^3^ Departamento de Biologia e Zootecnia, Universidade Estadual Paulista, Ilha Solteira, São Paulo 15385-000, Brazil; ^4^ Department of Biology, The Pennsylvania State University, University Park, PA 16803, USA; ^5^ Department of Biodiversity and Aquaculture Center, Universidade Estadual Paulista, Rio Claro, São Paulo 13506-900, Brazil; ^6^ Sistema Nacional de Investigación, SENACYT, City of Knowledge, Clayton, Panama, Republic of Panama; ^7^ Department of Biology, University of Massachusetts Boston, Boston, MA 02125, USA; ^8^ New York University Abu Dhabi, Abu Dhabi, UAE; ^9^ One Health Microbiome Center, Center for Infectious Disease Dynamics, Ecology Institute, Huch Institutes of the Life Sciences, The Pennsylvania State University, University Park, PA 16803, USA

**Keywords:** *Boana faber*, Brazil, co-infection, co-occurrence network analysis, deforestation, nematode

## Abstract

Habitat fragmentation can negatively impact wildlife populations by simplification of ecological interactions, but little is known about how these impacts extend to host-associated symbiotic communities. The symbiotic communities of amphibians play important roles in anti-pathogen defences, particularly against the amphibian chytrid fungus *Batrachochytrium dendrobatidis* (*Bd*). In this study, we analyse the role of macroparasitic helminth communities in concert with microbial communities in defending the host against *Bd* infection within the context of forest fragmentation. We found that skin microbial and helminth communities are disrupted at fragmented habitats, while gut microbiomes appear more resilient to environmental change. We also detected potential protective roles of helminth diversity and anti-pathogen microbial function in limiting *Bd* infection. Microbial network analysis revealed strong patterns of structure in both skin and gut communities, with helminths playing central roles in these networks. We reveal consistent roles of microbial and helminth diversity in driving host–pathogen interactions and the potential implications of fragmentation on host fitness.

## 1. Introduction

Current estimates indicate that parasites comprise around 40% of global biodiversity and are involved in nearly 75% of all trophic linkages in food webs [1,2]. Consequently, more diverse ecosystems with greater numbers of trophic linkages should exhibit higher parasite diversity, and parasite diversity can be seen as an indicator of overall biodiversity and ecosystem health [3–6]. A higher diversity of less harmful parasites may also reduce the impacts of more harmful parasites on hosts by priming immune responses [4,7–9]. Helminths generally have a tighter coevolutionary history with their hosts than many microbial parasites, making their infection a more established part of the host’s immune system development [10,11]. For this reason, infection with diverse assemblages of parasites could train immune responses and reduce the severity of inflammatory responses during subsequent infections [7,8,10]. Habitat loss and fragmentation are major threats to complex symbiotic interactions, contributing to coextinctions of both hosts and their symbionts [12–15]. Thus, ecosystem disturbances caused by habitat fragmentation could disrupt helminth and microbial communities to the detriment of their hosts.

The amphibian chytrid fungus *Batrachochytrium dendrobatidis* (*Bd*) is a globally distributed parasite of amphibians and can be highly pathogenic in hundreds of susceptible host species [16]. However, the impacts of coinfections with helminths and *Bd* on amphibian hosts are poorly understood, especially in the context of habitat fragmentation. The factors modulating the effect of habitat fragmentation on parasite infection dynamics are complex, but some possible mechanisms that may exacerbate disease risk include increased population density concentrating susceptible hosts, reduced community diversity resulting in more interactions between susceptible hosts and pathogens, and increased stress on hosts weakening their immune function [14,17–22]. Fragmentation-driven simplification of ecological systems would thus have strong downstream effects on co-occurring mutualistic and parasitic communities [13,23–25]. The host-associated microbiome plays an important role, in conjunction with the innate immune system, helping to protect hosts from pathogen colonization or growth [26–28]. Recent work has revealed that certain bacteria naturally present in the amphibian skin microbiome can inhibit fungal growth, providing a valuable metric of microbiome function [29–31]. The outcome of pathogen infection may thus be determined by immune priming from helminth infection jointly with adaptive shifts in the microbiome through enrichment with key anti-pathogen members, both resulting in bolstered immune defences [32–35].

While the microbiome is a source of anti-pathogen protection for hosts, this community can also be the source of pathogenesis when imbalanced, leading to a state called dysbiosis [36–38]. This can happen when beneficial bacteria are lost, harmful bacteria increase or community composition is altered [33,37–39]. Similarly, when helminth communities become imbalanced, individual taxa may become overly abundant and pathogenic [40,41]. Within this context, greater community variation may indicate dysbiosis [38], though identifying community dysbiosis can be challenging as it can vary between different hosts and systems. Shifts in the amphibian skin microbiome and loss of microbial diversity have been linked to infection by more pathogenic types of helminths, suggesting a cascading influence of helminth community disruption on microbiome stability [38,42]. Other research has found that habitat loss can disrupt amphibian microbiomes [43,44] and helminth communities [45–47], indicating the potential for negative impacts on host fitness despite clear signs of disease.

To test how forest fragmentation alters the interplay between helminth communities, host microbiomes and *Bd* infection, we conducted a study using the forest-associated treefrog *Boana faber* in the state of São Paulo, Brazil. We surveyed the helminth communities, skin and gut microbiomes and *Bd* infection status of *B. faber* across six sites, three forest fragments and three areas of continuous forest. We predicted that frogs from continuous forest habitats would have more species-rich parasite communities, with parasite, skin microbiome and gut microbiome assemblages that are more similar among sites. Additionally, we predicted that frogs in fragmented habitats would have less species-rich parasite communities with the parasite, skin microbiome and gut microbiome assemblages that are more variable among sites. Finally, we predicted that frogs with relatively lower helminth and microbiome diversity would display elevated *Bd* infection loads, and this effect was anticipated to be more pronounced in fragmented habitats. Our findings provide insights into the complex interplay between amphibian microbiomes, helminth communities and *Bd* infection and highlight the importance of endemic parasites in amphibian health.

## 2. Methods

### 2.1. Field methods

**Figure 1.**
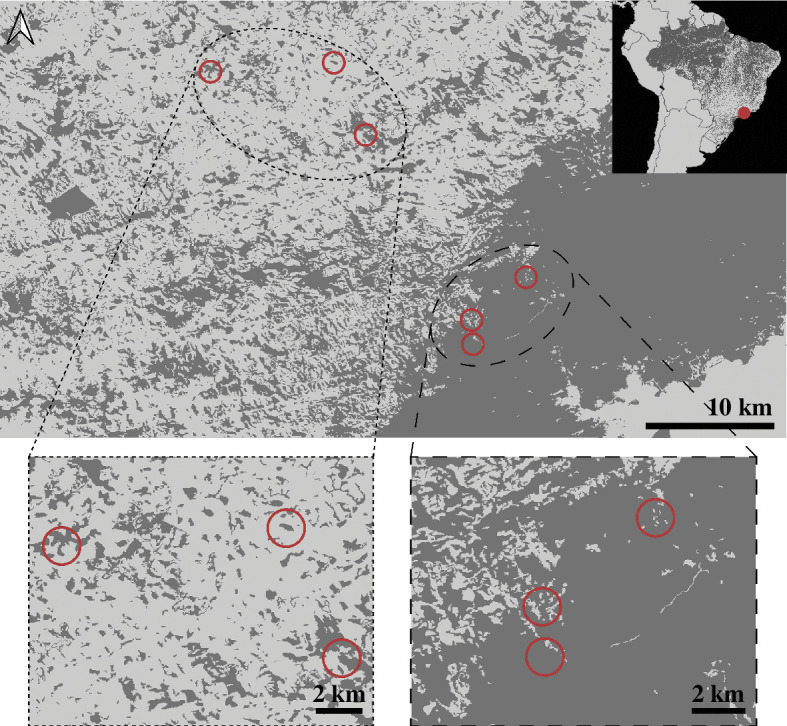
Map showing forested (dark grey) and deforested (light grey) areas in the study region. The distribution of sampling sites is represented by red circles within the fragmented forest (dotted line) and continuous forest (dashed line) sites.

We collected individuals of *B. faber* in São Luiz do Paraitinga in the state of São Paulo, Brazil, during the rainy/breeding season of 2021. Our study sites were spread across a section of the expansive Brazilian Atlantic Forest and the bordering mosaic landscape of forest fragments and farmland (figure 1). We captured five frogs across each of three forest fragments (*n* = 15) and three sites within a continuous forest (*n* = 15). Of important note, we had limited sample sizes owing to the nature of this study requiring animal euthanasia, so results should be interpreted with that understanding. We transported frogs back to the field laboratory in clean plastic bags then swabbed, weighed and measured them. We handled each frog with new gloves and rinsed frogs with distilled water before swabbing to remove debris and transient microbes. Using a sterile rayon swab (Medical Wire), we swabbed 10 strokes down the axilla and oblique of each frog and five strokes on the underside of each foot [48]. We collected an additional field control swab by swabbing a clean glove sprayed with distilled water after each group of frogs was processed (*n* = 3) to detect contamination in the distilled water or on gloves. We stored swabs at −20°C until DNA extraction. We euthanized frogs using lidocaine applied to the ventral surface. We sampled the gut microbiome by removing a 2 cm length of the upper small intestine, which we stored in RNAlater at −20°C until DNA extraction. We thoroughly searched all internal organs for metazoan parasites under dissecting scopes. Any recovered parasites were temporarily stored in 0.7% saline solution, then fixed in alcohol-formol-acetic acid and stored in 70% ethanol. For parasite identification, cestodes and acanthocephalans were stained with Semichon’s carmine and cleared with clove oil. Nematodes were cleared with lactophenol and examined using temporary mounts. We identified helminths by comparing key morphological features to available keys and original species descriptions [49,50].

### 2.2. Bacterial culturing

To obtain a metric of the *Bd*-inhibitory potential of the microbiome of this host species, we used swabs taken from *B. faber* from the same sampling locations in 2020. These swabs were stored in 1 mL of sterile cryomedia (1% tryptone and 20% glycerol) and kept at −20°C until culturing. We thawed and vortexed tubes containing cryomedia to evenly suspend bacteria before plating 20 µl on R2A agar. The liquid was spread using sterile spreader bars to evenly distribute bacterial cells over the plate. We incubated each plate at 21°C for up to 2 weeks until colony growth plateaued. As they appeared, we picked unique colonies and re-streaked them onto fresh R2A agar to confirm purity prior to cryopreservation.

To collect bacterial secondary metabolites for use in *Bd* inhibition assays, 10 µl of cryopreserved culture was added to 3 ml of 1% tryptone broth in 5 ml borosilicate culture tubes. Cultures were incubated on a shaker for 72 h at room temperature to allow depletion of nutrients in the media. Bacterial suspensions were transferred to 2 ml microcentrifuge tubes and centrifuged at 7500×*g* for 10 min to pellet bacteria. The supernatant was decanted and filtered through a 0.22 µm syringe filter prior to freezing at −20°C for storage until inhibition assays commenced. We conducted inhibition assays following previously described methods [51,52]. Briefly, we grew *Bd* in 96-well culture plates with bacterial metabolites and monitored growth over time. If *Bd* growth was inhibited compared with controls, we considered the bacteria producing the given metabolite to be *Bd* inhibitory.

### 2.3. Molecular methods

After fieldwork was completed, we extracted DNA from all skin swabs and gut fragments at the State University of São Paulo, Rio Claro, SP, Brazil using Qiagen DNeasy Blood and Tissue kits, following the manufacturer protocol. We included an extraction control consisting of extraction reagents with no sample added to monitor possible contamination during the extraction process. Eluted DNA was sent to the University of Alabama, Tuscaloosa, AL, USA for further molecular analysis. We used quantitative PCR (qPCR) assays to detect *Bd*, diluting DNA 1:10 and quantifying *Bd* loads using Taqman qPCR assays which target the ITS1 and 5.8S gene region [53] and gBlock synthetic *Bd* standards diluted from 10^6^ to 10^2^ gene copies (gc). We ran plates in duplicate and ran mismatching samples on a triplicate plate. Only samples that were positive on two plates were recorded as positive for analyses. We averaged *Bd* loads across the duplicate plates and, for analyses, divided by host mass to control for differences in host body size. We further log10-transformed load values to account for non-normal distributions characteristic in pathogen load data.

To identify bacterial isolates, we picked single colonies from pure cultures and placed them in 8-strip tubes with 50 µl sterile MilliQ water. After vortexing, we added 5 µl of bacterial suspension to 96-well plates containing 100 µl Chelex 100 solution (5 g Chelex 100/50 ml MilliQ) for DNA extraction. We heated the plates to 99°C for 20 min in a thermocycler, then stored at 4°C for 48 h. Next, we centrifuged plates at 3900 rpm for 2 min and aliquoted 2 µl of supernatant to a clean 96-well PCR plate. We amplified extracted DNA using 16S rRNA primers 907R and 8F. We sequenced reverse strands at MCLab (San Francisco, CA, USA) and trimmed reads using Geneious Prime.

To metabarcode microbial communities from skin swabs, we followed the Earth Microbiome Project 16S Illumina Amplicon Protocol [54,55], which targets the V4 region of the prokaryote *16S* rRNA gene using a dual-index approach with 515F and 806R barcoded primers. PCR-amplified DNA extracted from skin swabs in duplicate plates using the following recipe per sample: 12.2 µl of UltraPure water, 4 µl of 5 × Phire reaction buffer (Thermo Scientific), 0.4 µl of 10 mM dNTPs (Invitrogen), 0.4 µl of Phire Hot Start II DNA Polymerase (Thermo Scientific), 0.5 µl each of 10 µM barcoded forward and reverse primers (Integrated DNA Technologies) and 2 µl of sample DNA. We ran duplicate PCR plates on SimpliAmp thermal cyclers (Thermo Scientific) according to the following protocol: 98°C for 3 min, 38 cycles of 98°C for 5 s, 50°C for 5 s, and 72°C for 15 s, then 72°C for 3 min before holding at 12°C. We included a negative control (water without template DNA) in each plate to monitor any potential contamination of PCR reagents. We combined duplicate plates and visualized amplicons in 1% agarose gel to confirm DNA amplification, which revealed fairly even amplification among samples. We pooled 2 µl of each sample into a single amplicon library, then purified the library using a QIAquick Gel Extraction Kit (Qiagen), then measured amplicon library concentration using the Qubit 2.0 fluorometer with a dsDNA Broad-Range Assay Kit (Invitrogen). The concentration of the purified library was 332.3 nM (80.8 ng/µl). The *16S* library was sequenced using Illumina MiSeq V2 with 2 × 250 bp paired-end reads at Tufts University Core Facility (TUCF Genomics), Boston, MA, USA. All microbiome sequences are uploaded to the NCBI Sequence Read Archive (BioProject PRJNA972709).

### 2.4. Bioinformatics

After receiving demultiplexed microbiome sequences, we imported forward and reverse reads for each sample into Quantitative Insights into Microbial Ecology II (QIIME2 version 2021.11). We used QIIME2 to generate amplicon sequence variant (ASV) tables and extract metrics of alpha and beta diversity for prokaryote microbiomes. Before analysing sequence data, we used the deblur pipeline to trim sequences to 250 bp based on quality scores and clustered sequences into ASVs. We used the SILVA 138 classifier to assign taxonomy to our sequence variants at 99% sequence similarity then removed chloroplast and mitochondrial sequences. One field control swab had a high number of reads, so we used the microdecon method of decontamination [56] to remove potential contaminants, which resulted in the removal of 262 ASVs. We then rarefied the ASV table to 3000 reads based on rarefaction curves (electronic supplementary material, figure S1), resulting in two of 60 samples being excluded, both of which were gut samples. For analyses of alpha diversity, we calculated the ASV richness for each sample. For analyses of beta diversity, we calculated Bray–Curtis and Jaccard distances between samples for skin and gut microbiomes separately. The Bray–Curtis metric of dissimilarity includes the relative abundance of microbial taxa based on sequence reads, while the Jaccard metric only includes the presence/absence of taxa. We used the first principal coordinate axis (PCo1) for each metric in analyses. We also calculated beta dispersion (partitioned by habitat) using the Bray–Curtis distance matrices, which measure the relative distance from the centroid of each sample in multidimensional space (betadisper function from vegan package in program R version 4.2.2) [57]. After sequencing anti-fungal bacterial isolates, we imported sequences into QIIME2 and clustered them into ASVs using vsearch at 99% sequence similarity. From the 76 inhibitory isolates, we identified 15 unique ASVs (electronic supplementary material, table S1). We then used ASV identity to calculate the proportion of sequence reads matching *Bd*-inhibitory isolates in skin microbiome samples (proportion *Bd*-inhibitory). Using helminth count data per sample, we calculated helminth infection intensity (log10-transformed), and helminth taxonomic diversity using the taxondive function in the vegan package [58]. Like the microbiome community analyses, we calculated Bray–Curtis and Jaccard dissimilarity between samples based on the helminth community and used PCo1 in analyses.

### 2.5. Statistical analyses

All analyses were run in R version 4.2.2 [57], and the code used to run analyses and create visualization is available as electronic supplementary material (Supplemental code 1). To test the effect of forest cover (continuous versus fragmented) on host body size (Mass and SVL), pathogen prevalence, pathogen loads and microbiome richness, we used the glmmTMB package [59], maintaining the sample site as a random effect. For host body size metrics, we used a Gaussian distribution. For *Bd* prevalence, we used a binomial distribution and logit-link function. For *Bd* infection loads, we used a negative binomial distribution with a zero-inflated formula to account for the inclusion of uninfected samples. For microbiome richness (skin and gut), we used a Poisson distribution. R code for these models can be found in the supplemental materials. Host body size, pathogen infection, parasite intensity and microbial richness metrics are given as mean ± standard deviation unless otherwise stated.

Using permutational analysis of variance (PERMANOVA; adonis function in vegan package) [60] on Bray–Curtis and Jaccard dissimilarities, we tested for differences in the skin microbiome community, gut microbiome community and helminth community between frogs from continuous and fragmented sites. We also tested differences in these three communities between frogs infected and uninfected with *Bd*, controlling for habitat using the ‘strata’ argument. To visualize differences in these communities, we plotted principal coordinate axes in ggplot2 [61].

We used the linear discriminant analysis effect size (LEfSe) method on the galaxy platform [62] to test for differentially abundant bacterial ASVs and helminth species between frogs at continuous and fragmented forest sites. We maintained default parameters. Using the heatmap.2 function from the gplots package in R [57,63], we created a plot to visualize differentially abundant taxa based on the LEfSe results.

We conducted a generalized linear model selection using the dredge function in the MuMIn package [64] to parse out key variables explaining *Bd* infection loads across samples. We included *Bd* negative and positive individuals for this step to avoid analysing different subsets of data in subsequent models. We included the following predictors to each model: skin and gut ASV richness, skin and gut microbiome dispersion, skin proportion of *Bd*-inhibitory bacteria, helminth infection intensity, helminth taxonomic diversity and habitat fragmentation. We selected the model with the lowest AICc score (electronic supplementary material, table S3). We used a generalized linear mixed model with a zero-inflated negative binomial distribution to run the best model, including the sample site as a random effect.

We computed correlation-based networks to compare patterns of ASV co-occurrence among host skin and gut microbial communities using the igraph package in R [65]. In these networks, each point (node) represents an ASV and connections (edges) between nodes represent significant pairwise correlations, which can be inferred as co-occurrences (when *r* is positive) or antagonistic interactions (when *r* is negative). We characterized network structure based on the following metrics: modularity, clustering coefficient, the average path length, graph density and average degree. These metrics allow for the characterization of the interconnectedness (average path length, graph density and average degree) and degree of clustering (modularity and average clustering coefficient) among ASVs within co-occurrence networks. We also calculated metrics of centrality for each node (betweenness and closeness) to characterize the placement of each node within the network. Higher values of betweenness centrality indicate nodes that lie at the connection of many other nodes, while higher values of closeness centrality indicate nodes that are near many other nodes. To construct networks, we first subsetted the data to remove ASVs comprising less than 0.02% of reads for gut and skin databases separately. This filtering step was performed to reduce the noise within networks owing to numerous rare taxa and resulted in 446 microbial taxa for skin networks and 246 microbial taxa for gut networks. To account for the innate differences between the microbial communities of the skin and gut, we built separate networks for each of these sources. For each microbial source, we calculated networks for each habitat type (continuous or fragmented) separately, both habitat types combined, and combined habitats including helminth taxa. This resulted in a total of eight networks (four for skin and four for gut). We built networks using statistically significant (adjusted *p* < 0.05) Pearson correlations between ASVs and with a correlation coefficient of less than −0.6 or greater than 0.6. We calculated the correlation between ASVs using the function rcorr from the package Hmisc [66] and the adjusted *p* values using the function *p*.adjust following the Benjamini and Hochberg method [67].

To test whether observed networks differed from random patterns of ASV co-occurrences, we generated 1000 random networks for each of the eight networks, using the same numbers of nodes and edges for the given network following the Erdös–Réyni model [68]. In these random networks, every possible edge between ASVs has an equal probability of occurring. We computed the random networks using the erdos.renyi.game function (with argument ‘type’ = ‘gnm’) from the igraph package. Topology metrics for each of the eight random networks generated represent the average and standard deviation (s.d.) of the 1000 networks. Density and average degree metrics were equal between observed and random values because these metrics are determined by the number of nodes and edges in each network, which were the same for both types of networks.

## 3. Results

Average *Bd* prevalence was not significantly different between sites (*z* = −0.81, *p* = 0.418), but we observed a tendency for higher prevalence at continuous (60 ± 40%) than fragmented (40 ± 35%) sites. Similarly, *Bd* loads ranged from 0 to 743 370 gc, with a mean of 59 679 ± 27 432 gc and tended to be higher at continuous (92 349 ± 52 785 gc) than fragmented (27 009 ± 13 373 gc) sites but these results were not statistically significant (*z* = 0.39, *p* = 0.699). Frogs were larger at continuous forest sites (mass = 68.0 ± 11.6, SVL = 96.8 ± 5.2) than fragmented sites (mass = 25.2 ± 17.4, SVL = 66.6 ± 16.5; mass: *z* = −4.30, *p* < 0.001; SVL: *z* = −4.76, *p* < 0.001; electronic supplementary material, figure S2).

Following all filtering and decontamination steps, we recorded 4334 ASVs across all samples. The dominant phyla in skin samples were Proteobacteria, Actinobacteriota, Bacteroidota, Planctomycetota, Acidobacteriota and Verrucomicrobiota and the dominant phyla in gut samples were Proteobacteria, Firmicutes, Planctomycetota and Euryarchaeota (electronic supplementary material, figure S3). We recorded fewer ASVs at continuous versus fragmented sites in the gut microbiome (62 ± 44 versus 97 ± 54, respectively; *z* = 7.11, *p* < 0.001) but similar numbers of ASVs in the skin microbiome (330 ± 121 versus 296 ± 126, respectively; *z* = −0.61, *p* = 0.542). Microbial diversity in gut communities (81 ± 52) was generally lower than in skin communities (313 ± 122). We sampled the small intestine for gut microbial community analyses, potentially leading to reduced diversity given that most gut microbes occur in the large intestine [69].

We recovered nine helminth species and morphotypes from the taxa Acanthocephala (*n* = 1), Cestoda (*n* = 1) and Nematoda (*n* = 7; table 1). Of the nematodes recovered, four different families were represented: Cosmocercidae (Cosmocercidae gen. sp., *Oxyascaris similis*, *Raillietnema* sp., *Rhabdias* sp.), Molineidae (*Oswaldocruzia subauricularis*), Onchocercidae (*Ochoterenella* sp.) and Physalopteridae (*Physaloptera* sp.). Two taxa (*Oxyascaris similis* and *Raillietnema* sp.) were only detected in frogs from sites within continuous forest.

**Table 1.** Helminth diversity recovered including number of frogs infected by each species, total infection intensity and mean infection intensity ± s.d.

taxa	*n* infected (prevalence)	total intensity	mean intensity ± SD
Cosmocercidae gen. sp.	18 (60.0%)	80	2.6 ± 5.47
*Ochoterenella* sp.	3 (10.0%)	10	0.33 ± 1.32
*Oswaldocruzia subauricularis*	10 (33.3%)	33	1.10 ± 2.32
*Oxyascaris similis*	9 (30.0%)	49	1.63 ± 3.02
*Physaloptera* sp.	1 (3.3%)	7	0.23 ± 1.28
*Raillietnema* sp.	2 (6.7%)	826	27.53 ± 105.88
*Rhabdias* sp.	7 (23.3%)	12	0.40 ± 0.93
Acanthocephala	1 (3.3%)	1	0.03 ± 0.18
Cestoda	1 (3.3%)	18	0.60 ± 3.29

**Figure 2.**
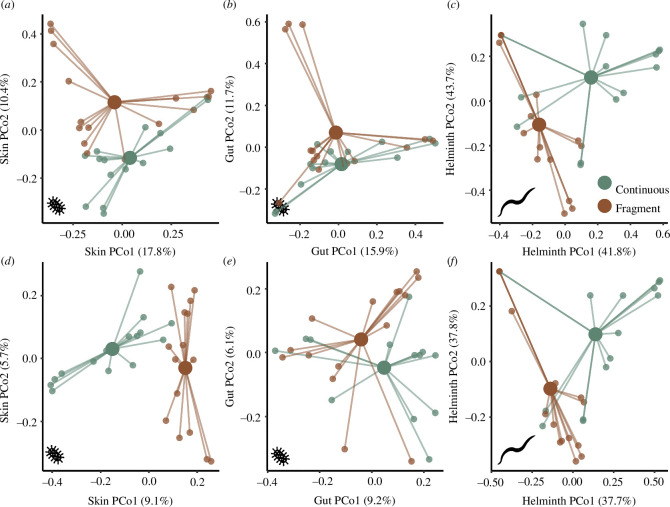
Spider plots showing similarity of skin microbiome communities (*a*,*b*,*d*,*e*) and helminth communities (*c*,*f*) for frogs at continuous (green) and fragmented (brown) sites. Dissimilarity is shown based on Bray–Curtis (*a*–*c*) and Jaccard (*d*–*f*) metrics. Large points with connecting lines represent group centroids.

Through PERMANOVA, we found a statistically significant difference in skin microbiome composition between continuous and fragmented sites considering both the presence/absence (figure 2*d*; electronic supplementary material, table S2) and relative abundance (figure 2*a*; electronic supplementary material, table S2) of microbial taxa. We did not find differences in gut microbiome composition between habitats using either metric (figure 2*b,e*; electronic supplementary material, table S2). We also found a statistically significant difference in helminth community composition between continuous and fragmented sites considering both the presence/absence (figure 2*f*; electronic supplementary material, table S2) and relative abundance (figure 2*c*; electronic supplementary material, table S2) of taxa. We found no difference in skin and gut microbiome composition between *Bd*-infected and *Bd*-uninfected individuals (electronic supplementary material, table S2).

Using LEfSe analysis, we detected 45 bacterial taxa and one helminth species as differentially abundant between samples from continuous and fragmented forest sites (electronic supplementary material, figure S4 and table S3). Most differentially abundant bacteria were from skin microbiome samples (*n* = 39), and 22 were higher in abundance at continuous forest sites. Six bacterial taxa were differentially abundant in the gut microbiome, two of which were higher in abundance at continuous forest sites. The nematode *Ox. similis* was more abundant at continuous forest sites. *Raillietnema* sp. was detected in two individual hosts, thus despite only occurring at continuous forest sites, this taxon was not detected as differentially abundant by LEfSe analysis.

**Figure 3.**
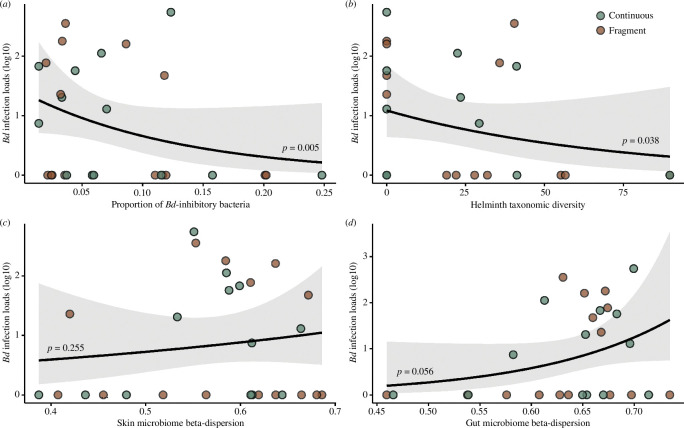
Scatterplots showing relationships between *Bd* infection loads (mass and log10-transformed) and proportion of Bd-inhibitory bacterial ASVs (*a*), helminth taxonomic diversity (*b*), skin microbiome dispersion (*c*), and gut microbiome dispersion (CD). Correlations are based on negative binomial relationships. Point colours indicate habitat type, with continuous forest in green and fragmented forest in brown. Shaded regions around lines show 95% confidence of fit.

After AICc model selection, the best model predicting *Bd* infection contained gut microbiome dispersion, proportion of *Bd*-inhibitory bacteria and helminth taxonomic diversity (electronic supplementary material, table S4). We found negative associations between *Bd* infection loads and proportion of *Bd*-inhibitory bacteria (*β* = −12.45 ± 5.11, *z* = −2.43, *p* = 0.015; figure 3*a*) and helminth taxonomic diversity (*β* = −0.02 ± 0.01, *z* = −1.99, *p* = 0.047; figure 3*b*). We also found a positive association between *Bd* infection loads and gut microbiome dispersion (*β* = 10.51 ± 5.34, *z* = 1.97, *p* = 0.049; figure 3*c*; electronic supplementary material, table S5).

**Figure 4.**
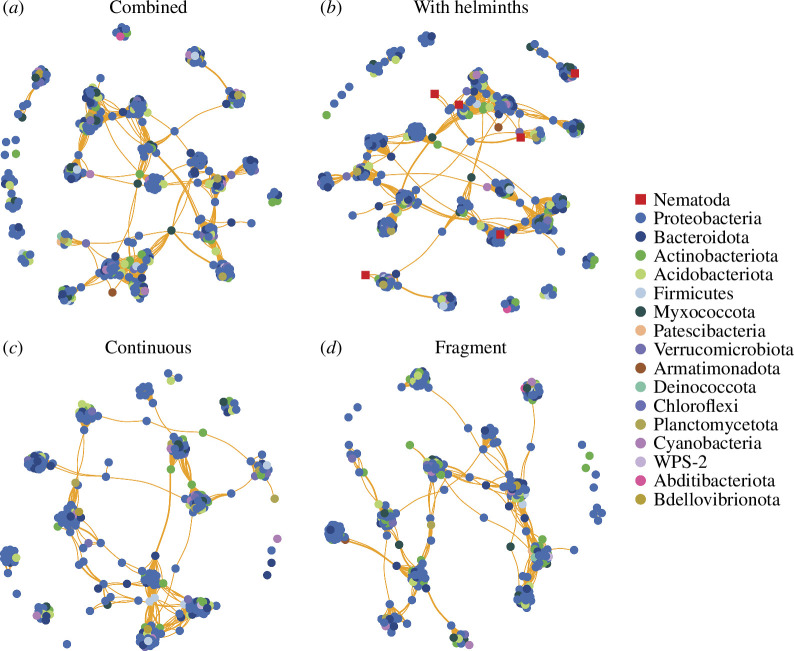
Skin microbiome co-occurrence networks showing co-occurrences among microbial and helminth taxa. Co-occurrence is calculated using Pearson correlations (distance cutoff *r* > 0.6 and < −0.6; *P* < 0.05) and the Fruchterman-Reingold layout. Microbiomes are shown as both habitat types combined (*a*), both habitat types combined and with helminth taxa included (*b*), for continuous forest samples only (*c*), and for fragmented forest samples only (*d*). Colored points indicate nodes (ASVs), and lines indicate edges (positive [orange] or negative [blue] correlations). Edge lengths are a function of layout and are not biologically meaningful.

**Figure 5.**
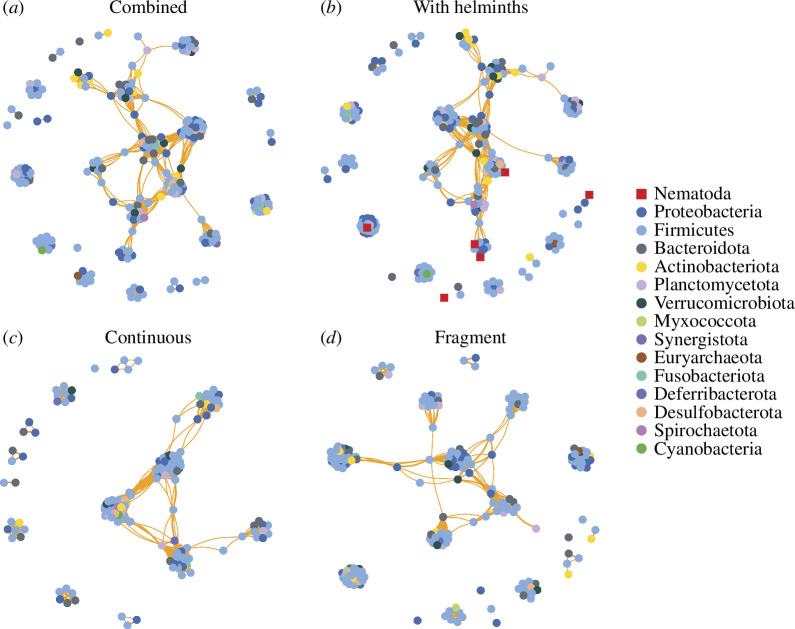
Gut microbiome co-occurrence networks showing co-occurrences among microbial and helminth taxa. Co-occurrence is calculated using Pearson correlations (distance cut-off *r* > 0.6 and < −0.6; *p* < 0.05) and the Fruchterman–Reingold layout. Microbiomes are shown as both habitat types combined (*a*), both habitat types combined and with helminth taxa included (*b*), for continuous forest samples only (*c*), and for fragmented forest samples only (*d*). Coloured points indicate nodes (ASVs), and lines indicate edges (positive (orange) or negative (blue) correlations). Edge lengths are a function of layout and are not biologically meaningful.

We analysed community interactions within host microbiomes using co-occurrence networks based on significant pairwise correlations (both positive and negative) among ASVs. We did not detect any negative (antagonistic) correlations between taxa in any network. Metrics of clustering (modularity and clustering coefficient) were high across all networks, indicating that microbial communities are cohesively structured into groups of cooccurring taxa. Specifically, modularity was ~5× higher and the clustering coefficient was ~13× higher than random networks (table 2). Comparing microbial sources, skin microbial communities had larger networks (table 2; figure 4) relative to gut networks (table 2; figure 5), driven by the greater number of taxa in the skin microbiome. Modularity, which quantifies clusters of associated taxa, was lower in the gut networks (compared with skin) when analysed separately by continuous and fragmented forest sites (table 2). Metrics of interconnectedness (average path length, graph density and average degree) were generally higher (at least ~2× higher) in skin networks compared with gut networks, likely driven by the greater number of taxa in these networks. Comparing between networks, the average path length was lower in the gut microbiome of frogs at continuous forest sites than in other gut networks (table 2), which could indicate a denser and more interconnected network. Graph density was higher when networks were split by habitat type (continuous or fragment), regardless of source (gut versus skin; table 2), which is driven by the loss of some taxa (vertices) in the smaller datasets. For gut networks, the average degree was uniformly higher when split by habitat type (table 2), indicating more densely connected networks, which is also likely driven by the loss of some taxa (vertices). For skin networks, the average degree was only higher for frogs at fragmented forest sites (table 2). Comparing metrics of centrality, *Ochoterenella* sp. had similar betweenness centrality relative to the average value for bacteria for skin and gut networks, while *Ox. similis* only had similar betweenness centrality values for the skin network (electronic supplementary material, table S6). This suggests that these taxa are well integrated into the microbial community. Cosmocercidae gen. sp. had similar closeness centrality in both skin and gut networks, while Archaea and *Ox. similis* had high values only in the gut network (electronic supplementary material, table S6), suggesting that these taxa are associated with clusters of taxa but potentially not as integral in driving cooccurrence.

**Table 2.** Topology metrics from microbial co-occurrence networks using Pearson correlations (distance cutoff *r* > 0.6 and < −0.6; *p* < 0.05). Column names indicate the following metrics: Ad, average degree; Apl, average path length; Cc, clustering coefficient; E, number of edges; Gd, graph density; Md, modularity; Nd, network diameter; V, number of vertices. Random networks were constructed using the given number of edges and vertices are displayed for each model.

source	network	size			clustering		interconnectedness		
		E	V	Nd	Md	Cc	Apl	Gd	Ad
skin	combined	4463	439	13	0.87	0.92	5.53	0.05	20.33
	*random*			4	0.16	0.05	2.32		
	continuous	3116	297	15	0.85	0.91	5.77	0.07	20.98
	*random*			3	0.16	0.07	2.14		
	fragment	4069	333	12	0.87	0.92	5.71	0.07	24.44
	*random*			3	0.14	0.07	2.08		
	helminths	4533	445	13	0.87	0.92	5.54	0.05	20.37
	*random*			4	0.16	0.05	2.33		
gut	combined	1744	239	10	0.83	0.93	3.69	0.06	14.59
	*random*			4	0.19	0.07	2.32		
	continuous	1333	145	8	0.69	0.94	2.92	0.13	18.39
	*random*			3	0.17	0.13	1.96		
	fragment	1853	200	9	0.78	0.95	3.9	0.09	18.53
	*random*			3	0.16	0.09	2.06		
	helminths	1804	245	11	0.83	0.92	3.73	0.06	14.73
	*random*			4	0.17	0.06	2.33		

## 4. Discussion

In this study, we found associations between amphibian microbiome composition, helminth community diversity and *Bd* infection dynamics in the context of forest fragmentation. We detected a consistent influence of habitat fragmentation on skin microbiome and helminth community assemblages, indicating possible disruptions to microbial recruitment in fragments of natural forest. Interestingly, gut microbiomes were not impacted by habitat fragmentation in the same way, with higher diversity but no difference in composition at habitat fragments. Skin microbiomes are largely shaped by available environmental microbial reservoirs [70,71] and helminth communities are often dependent on transmission through the environment [72]. Conversely, gut microbiomes are relatively buffered from direct environmental influences and can be largely shaped by host-specific factors [73,74]. For these reasons, disruption of the biotic and abiotic environment characteristic in habitat fragments [13] likely has a greater influence on the skin microbial and helminth communities, while gut microbes remain more stable across habitats. The increased diversity of gut microbes at fragments may represent rare transient taxa as they do not influence the composition.

We found a relationship between the dispersion of the gut microbiome community, a metric of microbiome instability, and *Bd* infection loads. Gut microbes have no direct interaction with *Bd*, and higher dispersion could be an indicator of microbiome dysbiosis [38,44,75,76]; hence, this correlation could be a proxy for systemic impacts of *Bd* infection on hosts, leading to a disruption of the gut microbiome community [77]. We found a negative correlation between the proportion of *Bd*-inhibitory bacteria in the skin microbiome and *Bd* infection, indicating that anti-pathogen members could defend against pathogen colonization and growth. Further supporting this finding, the four frogs with the highest proportions of inhibitory bacteria in their microbiome (>15%) were uninfected. Two mechanisms could explain this relationship between pathogen infection and *Bd*-inhibitory properties of the skin microbiome; either standing defences in the microbiome could limit infection, or microbial recruitment during infection could allow for pathogen clearing. However, it is important to note that our dataset is correlational in nature and future experimental tests are required to support these hypotheses and elucidate mechanistic drivers.

Helminth infection may trigger similar immune pathways to *Bd* infection [10,78–80], particularly since many nematode species enter hosts through skin penetration [41]. We found a negative association between helminth diversity and *Bd* infection. It is plausible that in our system, infection by a diverse assemblage of helminth species is triggering physiological responses in the skin such as secretion of anti-microbial peptides [81], which in turn influences microbial recruitment [79]. In humans, helminth infection has been linked to reduced autoimmune diseases through modulation of regulatory T cells and anti-inflammatory pathways [82–84]. Thus, helminth infections, particularly those with skin-penetrating larval stages, could trigger a similar protective immune cascade. The combination of microbiome community modification and enhanced immune function is a potential mechanism linking helminth diversity with *Bd* infection in this system.

Our co-occurrence networks revealed consistently high patterns of clustering in both skin and gut microbial communities. This, along with the observation that all correlations between taxa were positive, indicates that the skin and gut microbiomes have strong cohesive structures in this system. Many taxa are likely dependent on the presence of other taxa to persist, and some taxa are potentially foundational for subsequent colonization by other community members [85]. The lack of negative correlations in our networks matches findings reported in a recent study in the same system [27] and could be partially influenced by our exclusion of rare taxa, which can have negative relationships simply owing to their appearance in a few samples [86]. Skin networks were larger than gut networks both in terms of the number of nodes and vertices, which is a result of the higher diversity of skin microbes. Clustering coefficients and graph density of gut networks are qualitatively higher than the same metrics in skin networks, although skin networks were more diverse overall. This suggests a simplified community in the gut, composed of fewer members that are consistently found across host populations with fewer transient bacterial taxa. Helminth taxa are likely shaping the microbiome, as seen in the centrality values of the taxa Cosmocercidae gen. sp., *Ochoterenella* sp., and *Ox. similis* in both skin and gut networks. These taxa appear to be important in stabilizing bacterial communities, acting as connection points in the networks and being proximally placed among many bacterial taxa. While the inclusion of helminth taxa does not change the network topology, interconnectedness or clustering in any meaningful way, it is important to remember that the microbiome community is associated with helminth taxa in both cases. One recent study in humans found that nematode infection was associated with disruptions to gut microbial networks [87], but the influence of complex helminth communities in relation to microbiome networks remains relatively unexplored. An experimental study comparing microbial networks between hosts that have intact or cleared helminth communities would be useful in determining the role of helminths in shaping host microbiomes.

Helminth communities were largely composed of various nematode taxa in this system, matching findings in other studies [88–90]. The known routes of infection for these species vary from ingestion of infective larvae (*Oxyascaris similis* and *Physaloptera* sp.), skin penetration by infective larvae (*Raillietnema* sp., *Oswaldocruzia subauricularis* and *Rhabdias* sp.), and vector transmission (*Ochoterenella* sp.) [91]. Therefore, these taxa are all highly dependent on environmental conditions for transmission, either directly or indirectly through intermediate hosts. Two nematode taxa (*Oxyascaris similis* and *Raillietnema* sp.) were completely absent from fragmented sites. Both of these taxa are environmentally transmitted, and therefore, may require more pristine habitats to persist. Some studies have discussed the utility of using helminths as indicators of ecosystem health owing to the reliance of many taxa on environmental conditions to persist [6,92–94]. Thus, these two taxa in this system may represent promising indicators of ecosystem health.

Interestingly, we found that frogs were consistently larger in both mass and body length at continuous forest sites. *B. faber* is a short-lived species [95] and engages in violent conspecific aggression during the breeding season [96,97], likely limiting the ability of smaller-sized males to engage in breeding behaviour. For these reasons, the observed differences in body size could represent an environmentally driven shift in this phenotype. Alternatively, adult lifespan may be reduced at habitat fragments, leading to younger cohorts of males comprising breeding groups.

Overall, we found evidence that both microbial and helminth diversity are integral parts of the host-pathogen response. These two symbiotic communities likely have synergistic influences on host defences, where helminth infection may shape microbial community composition and simultaneously bolster immune system function [10,98]. Forest fragmentation could impact the composition of both microbial and helminth communities owing to changes in environmental pools of microbes and helminth infective stages [70]. Healthy ecosystems are generally rich in species diversity and trophic linkages [4,6], and as parasite species rely on their hosts to persist, parasite diversity is a good indicator of total ecosystem health.

## Data Availability

Raw data used for analyses has been uploaded as supplemental files [99]. Any additional data are available on request. Microbiome sequence reads generated in this study have been uploaded to the NCBI Sequence Read Archive (BioProject PRJNA972709). R code used to run all analyses is included as a supplemental file.
